# A Hybrid Algorithm of ML and XAI to Prevent Breast Cancer: A Strategy to Support Decision Making

**DOI:** 10.3390/cancers15092443

**Published:** 2023-04-25

**Authors:** Fabián Silva-Aravena, Hugo Núñez Delafuente, Jimmy H. Gutiérrez-Bahamondes, Jenny Morales

**Affiliations:** 1 Facultad de Ciencias Sociales y Económicas, Universidad Católica del Maule, Avenida San Miguel 3605, Talca 3460000, Chile; fasilva@ucm.cl (F.S.-A.); jmoralesb@ucm.cl (J.M.); 2 Doctorado en Sistemas de Ingeniería, Facultad de Ingeniería, Universidad de Talca, Camino Los Niches Km 1, Curicó 3340000, Chile; hnunez@utalca.cl

**Keywords:** machine learning, explainable artificial intelligence, risk factors, breast cancer prevention, decision support systems

## Abstract

**Simple Summary:**

Breast cancer is one of the most common health problems in the world. As a result, governments and researchers in different countries are trying to help prevent the disease. In this work, we develop a clinical decision support methodology based on machine learning tools. This methodology helps identify breast cancer patients and determine the risk factors for this disease. In addition, the proposed strategy can help detect the disease in its early stages using modern easy-to-interpret machine learning tools.

**Abstract:**

Worldwide, the coronavirus has intensified the management problems of health services, significantly harming patients. Some of the most affected processes have been cancer patients’ prevention, diagnosis, and treatment. Breast cancer is the most affected, with more than 20 million cases and at least 10 million deaths by 2020. Various studies have been carried out to support the management of this disease globally. This paper presents a decision support strategy for health teams based on machine learning (ML) tools and explainability algorithms (XAI). The main methodological contributions are: first, the evaluation of different ML algorithms that allow classifying patients with and without cancer from the available dataset; and second, an ML methodology mixed with an XAI algorithm, which makes it possible to predict the disease and interpret the variables and how they affect the health of patients. The results show that first, the XGBoost Algorithm has a better predictive capacity, with an accuracy of 0.813 for the train data and 0.81 for the test data; and second, with the SHAP algorithm, it is possible to know the relevant variables and their level of significance in the prediction, and to quantify the impact on the clinical condition of the patients, which will allow health teams to offer early and personalized alerts for each patient.

## 1. Introduction

According to Sung et al. [1], in 2020, cancer was one of the main diseases of people in the world, with around 20 million cases and at least 10 million deaths. Undoubtedly, it is one of the main concerns of countries and health services. In addition and due to the COVID-19 pandemic, health services have become even more stressed (e.g., the case of oral cancer in India Gupta et al. [2], cancer diagnostic delay in northern and central Italy, cited in Ferrara et al. [3], and substantial increases in the number of avoidable cancer deaths in England, mentioned in Maringe et al. [4]), the aforementioned, and according to authors such as Spicer et al. [5], González-Montero et al. [6] in the area of cancer and other complex diseases.

On the other hand, different authors, such as Saini et al. [7], Nolan et al. [8], Collaborative et al. [9], and others, have indicated that the treatment of some cancer patients has been interrupted, and the pandemic has increased their negative consequences. In fact, Ricciardiello et al. [10] points out that due to waiting for treatment, deaths have increased by 12%. Therefore, it is dramatically important to timely detect and treat this type of disease in the population, that is, to anticipate complex patient situations. For this reason, COVID has taken a lot of time and attention from the clinical team, negatively impacting patients with other types of diseases, generating delays in care and in terms of confirming the diagnosis, as pointed out by the authors Picchio et al. [11], Radfar et al. [12], and others. For these reasons, using different methodologies and tools to prevent and detect this disease early is important, helping the clinical team and patients.

According to the World Health Organization (WHO) https://www.who.int/news-room/fact-sheets/detail/cancer (accessed on 15 February 2023), cancer is one of the leading causes of death worldwide, with almost 10 million deaths in 2020. In addition, the most common cancers are breast, lung, colon and rectal, and prostate cancer, of which about a third of deaths are due to tobacco use, high body mass index, alcohol use, low fruit and vegetable intake, and lack of physical activity. The most common in 2020 (in terms of new cases of cancer) were breast (2.26 million cases); lung (2.21 million cases); colon and rectum (1.93 million cases); prostate (1.41 million cases); skin (non-melanoma) (1.20 million cases); and stomach (1.09 million cases), and the most common causes of cancer death in 2020 were lung (1.80 million deaths); colon and rectum (916,000 deaths); liver (830,000 deaths); stomach (769,000 deaths); and breast (685,000 deaths). This quantification implies that the problem is so important that governments worldwide must continue working on actions and political measures to advance in this regard.

For our work, we are concentrating on breast cancer to consider the most important number of cases globally. Authors such as Garcia et al. [13], Chavez et al. [14] show that over 1.3 million cases of invasive breast cancer are diagnosed worldwide, and more than 450,000 women die from breast cancer annually. However, in the US, Chavez et al. [14] show that breast cancer has declined due to earlier detection due to improved adjuvant therapy and, more recently, decreased incidence due to decreased cancer rates.

Machine learning (ML) belongs to artificial intelligence, which focuses on machine learning from data. ML draws on different fields, ranging from statistics, mathematical algorithms, and data structures to deriving predictions and rules that support humans. ML has been applied in different areas, and its applications are very wide. They include, e.g., the prediction of the length of stay of cardiac patients in hospitals (Hachesu et al. [15]), classification of disease, cited by Saranya and Pravin [16] where the authors propose a sensitivity analysis for ML-based heart disease classification or instance, classifying chronic patients in risk for medical care using a lot of ML tools (Silva-Aravena et al. [17]), among many others in the health field.

The indicated strategies require the ability to interpret the predictions proposed by the algorithms, which is studied in a subfield of ML called explainable artificial intelligence (XAI) (see, for example, Madanu et al. [18], Loh et al. [19], Panigutti et al. [20]). Interpretability can significantly influence the decision to use a particular model; researchers can use a simpler model for complex problems or use ones requiring less computing power. In addition, justifying specific results that can produce valuable information that people can analyze and understand to produce knowledge and help better decision making.

Regarding the early detection of breast cancer, some authors in the world have been working with some sophisticated techniques and methodologies, such as the machine learning approach and others (see., e.g., Osareh and Shadgar [21], Ahmad et al. [22], Yue et al. [23], Ganggayah et al. [24], Rajendran et al. [25], Ming et al. [26], Rajendran et al. [27], Chaurasia and Pal [28], Naji et al. [29], Rabiei et al. [30], Zeng et al. [31]), which has allowed them to help predict and classify different types of diagnoses, estimate the survival rate, and provide clinical follow-up to patients with breast cancer that facilitate decision making by the health team in the aid of patients. Some applications include the XAI algorithm, favoring health team knowledge and their decision making (see Idrees and Sohail [32], Rodriguez-Sampaio et al. [33]).

Other diverse strategies have been studied to manage breast cancer, such as machine learning (see, e.g., Nindrea et al. [34], Magna et al. [35,36], Yu et al. [37]), Delphi techniques (Iunes et al. [38]), spatial autocorrelation (Durán and Monsalves [39]), and medical techniques such as genetic epidemiology, chemotherapy, radiotherapy, telerehabilitation (see, e.g., Ramírez-Parada et al. [40], Zavala et al. [41], Mella-Abarca et al. [42], Valverde-Ampai et al. [43]), among others.

In our methodology presented in this paper, we propose a novel methodology to classify breast cancer using a machine learning algorithm embedded with an XAI technique for Indonesian patients. This strategy was developed so that health teams provide information and preventive actions to patients who have yet to develop the disease of breast cancer. In addition, the methodological proposal serves as input for physicians to address the best treatment for patients classified with the disease and with the diagnosis confirmed by clinical examinations. In both cases, the methodology is used only as a support strategy for the clinical actions implemented by the health teams in each case.

Our main contributions to this work are as follows: First, a benchmarking strategy that allows selecting the best ML model, using some indicators such as accuracy, precision, and recall to classify patients with and without breast cancer from a set of data on the reproductive health of Indonesian women, high-fat diets, and risk factors for body mass index. Finally, the second contribution is an automated and hybrid methodology based on an embedded ML schema with an XAI algorithm. The mix, ML + XAI, makes it possible to predict the state of each patient, follow prevention actions, and know which variables and how they can affect each patient’s medical condition.

This paper is organized as follows. Section 2 presents related literature concerning the techniques and methods used to manage breast cancer patients’ risk. Section 3 presents the main methodology used in our work. The results obtained from our strategy are presented in Section 4. A section of discussion is presented in Section 5. Finally, in Section 6, we draw conclusions and make suggestions for future work.

## 2. Related Literature

Below, we present how breast cancer is managed and prevented worldwide and how it has intensified with the pandemic. In addition, we show the various proposals for strategies in the state-of-the-art and the gaps that justify the choice of our proposed methodology.

### 2.1. Impact of COVID-19 for Managing Cancer in the World

For Cheng et al. [44], the cancer health problems of the population have been one of the main challenges of public policies, both in the clinical and budgetary spheres. According to Flores et al. [45], this challenge becomes more complex during the pandemic. This situation, such as the inability to manage the cancer health problems of patients, generates important gaps between the health demand and available resources (Obek et al. [46], Levit et al. [47], Abu-Odah et al. [48], Hwang et al. [49]).

Authors, such as Okereke et al. [50] and others, point out that the daily burden experienced by health services added to the demand for attention to increasingly complex problems, such as cancer and others (Elkaddoum et al. [51], Al-Quteimat and Amer [52]), and the reorganization of the medical supply as a result of the pandemic (see, e.g., Radfar et al. [12] ) generates an unavoidable problem: waiting lists in those processes, such as cancer, are not related to the health emergency caused by COVID-19 (see, e.g., Sorrentino et al. [53], de la Vina et al. [54], Cadili et al. [55]). In addition, Lo et al. [56] mention that the cancer waiting time is longer due to COVID-19 and that according to Greenwood and Swanton [57], 31,000 fewer patients started treatment for cancer across the UK between April and August 2020 in the pandemic period, compared with the same period in the previous year. Authors such as Sud et al. [58], Malagón et al. [59] point out that while cancer patients wait, health conditions worsen due to the risk of tumors progressing, and in extreme cases, waiting can cause their death.

So many authors, such as Vourganti et al. [60], Lu et al. [61], Janas [62], Keenan and Frizelle [63], before and after the pandemic, have been working on different methodologies, techniques, and tools for improving and detecting cancer episodes in patients. Other authors, such as Zhu et al. [64], Leung et al. [65], have developed applications to predict if patients will have cancer in the future.

### 2.2. Strategies for the Prevention and Management of Breast Cancer

Various strategies have been developed to manage cancer in health services. For instance, Adams et al. [66] developed a lung nodule management strategy that combines with an artificial intelligence malignancy-risk score, achieving savings per patient assessed. Others, such as Osareh and Shadgar [21], Yue et al. [23], Ming et al. [26], Chaurasia and Pal [28], Naji et al. [29], Rabiei et al. [30], Nindrea et al. [34,36], Santiago-Montero et al. [67], have used machine learning algorithms to predict breast cancer diagnosis. Addittionally, in the same line, Ahmad et al. [22], Zeng et al. [31] use machine-learning strategies to predict breast cancer recurrence. Other authors, such as Yerukala Sathipati and Ho [68], had intended to predict the disease’s different stages and proposed treatment strategies.

To concentrate on breast cancer, other authors have proposed different techniques related to mathematical models. For instance, Padmanabhan et al. [69], Jarrett et al. [70] use mathematical models for the dynamics of breast cancer and immune checkpoint inhibitors. Even Yang et al. [71] has developed and validated a mathematical model that predicts how glucose dynamics influence metabolism and, therefore, tumor cell growth. On the other hand, Szczurek et al. [72] presents theoretical grounds for the metastatic bottleneck with a simple stochastic model used for breast cancer survival. In addition, Avanzini et al. [73] developed a mathematical model of tumor evolution and shedding to predict the size at which it becomes detectable.

In other aspects, when patients do not receive prompt attention, the complexity of breast cancer increases, and the therapies sometimes cannot work. For this reason and shown by Chamseddine and Rejniak [74], modeling such complex systems and predicting how tumors will respond to therapies require mathematical models that can handle various types of information and combine diverse theoretical methods on multiple temporal and spatial scales, that is, through hybrid models. In the same way, Altaf [75] designed a hybrid model based on Pulse-Coupled Neural Networks and Deep Convolutional Neural Networks for breast cancer diagnosis. In addition, Hosseinpour et al. [76] presented a hybrid breast cancer risk assessment algorithm. For that, the fuzzy method obtains the tumor’s effect on breast cancer, and an improved Random Forest Classification predicts an overall breast cancer risk.

Other sophisticated types of strategies have been considered for breast cancer diagnosis, such as nature-inspired meta-heuristic optimization algorithms, presented by Oladele et al. [77], or a heuristic neural network and meta-heuristic models (see, e.g., Alsaeedi et al. [78], Kang et al. [79]). Finally, survival strategies are successfully designed and studied for breast cancer, where for instance Moncada-Torres et al. [80] and others compare different techniques for survival analysis, e.g., Cox proportional hazard; machine learning models for survival analysis; random survival forests; survival support vector machines; and extreme gradient boosting, which demonstrates a better performance of the cancer attention patients process.

### 2.3. Justification of the Chosen Method

In light of the background and the strategies presented in the state-of-the-art, it is clear that breast cancer prevention processes can be optimized through machine learning methods and interpretability strategies to support medical decision making and benefit patients. patients. As a result, the main findings that justify adopting the chosen method are presented below:

1. Extensive international evidence demonstrates the importance of including dynamic and machine-learning methodologies for the prevention and management of patients with breast cancer. That is why it is urgent in the countries with the highest incidence to implement these tools that support medical management to help patients.
2. One of the elements rarely addressed in the literature on breast cancer prevention is the inclusion of interpretable algorithms that facilitate understanding for decision makers.
3. Finally, one of the relevant factors discussed in the literature is the importance of medical opinion when defining methods, criteria, and factors that allow the development of the oncological strategy since each clinical unit and its committee have its way of managing its patients.

The conclusions reveal the importance of developing breast cancer prevention systems that support medical decision making and, in turn, provide each patient with relevant information for breast cancer prevention in a personalized and early way.

## 3. Materials and Methods

This section presents the methodology’s main elements for classifying breast cancer patients using ML + XAI.

### 3.1. New Strategy to Classify Patients with Breast Cancer

**Figure 1:**
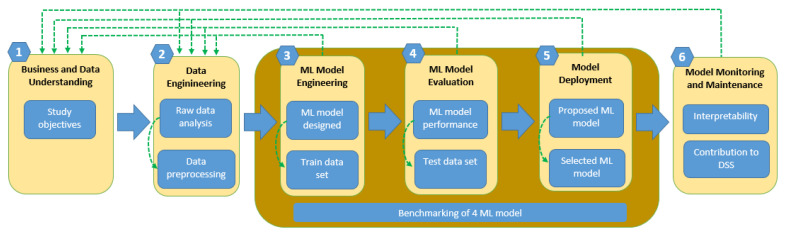
CRISP-ML (Q) mixed with an XAI algorithm to optimize decision-making for breast cancer prevention.

The structure of the patient classification strategy is based on the Intersectoral Standard Process for the development of Machine Learning applications with the quality assurance methodology (CRISP-ML(Q)), a method widely used in the health sector and which has been mentioned in different works, such as Silva-Aravena et al. [17], Kolyshkina and Simoff [81], Silva-Aravena and Morales [82], Silva-Aravena et al. [83]. Additionally, we have incorporated an explainability algorithm, XAI, into this strategy to provide better-quality information that favors clinical decision making. This hybrid method, ML + XAI, is adapted to improve the management of patients with breast cancer, strongly supported by the interpretability strategy. The main components of the methodology are presented in Figure 1.

For the particular case of this study, the methodology presents six stages: (1) the objective of the study is to determine a model of ML and XAI that allows for predicting the clinical condition of patients and provides an interpretation that supports the decision-making of the health team; (2) raw data preprocessing from anonymous patients; (3) evaluate different classification algorithms; (4) create a performance ranking of the models using the test data; (5) select the best ML model; and finally (6) use the XAI algorithm at the patient level that contributes to clinical decision support systems.

### 3.2. Case Study: Breast Cancer Patients in Indonesia

The data were encoded as part of the processing. We follow a label encoding strategy in the variables of one class; we use a one-hot encoding strategy in the variables of more than one class. As a result of this processing, in the dataset, 0 will indicate the absence of the feature and 1 its presence.

The case study used the public data of women from Indonesia with and without breast cancer (see, Nindrea et al. [84]) and was published (https://data.mendeley.com/datasets/xfcyrffhy7/2, accessed on 1 February 2023). Some risk factors, pointed out by [85], Alsolami et al. [86], Solikhah et al. [87], are included in the study case, such as age at menarche, the first pregnancy, age at menopause, and others. In addition, a high-fat diet and determinants of body mass index (BMI), parity, breastfeeding, and other factors for breast cancer in Indonesian women. The registries contain information on patients with and without breast cancer. The data were collected from the 1st June to 31 September 2020. Two hundred breast cancer patients and two hundred non-breast cancer patients in Indonesia provided the online survey. The study would help identify the potential risk to Indonesian women preventing breast cancer and women in other parts of the world.

### 3.3. Extreme Gradient Boosting: XGBoots Algorithm to Predict Breast Cancer

Multiple decision trees are sequentially combined in the ensemble learning technique known as XGBoost (see, e.g., Ramraj et al. [88], Tian et al. [89]). To represent a dataset with m features and n labels, let $D=\left(x_{i},y_{i}\right)(|D|=n,x_{i}\in R^{m},y_{i}\in R^{n})$ be used. Using XGBoost’s *j*th decision tree, a sample $\left(x_{i},y_{i}\right)$ is predicted by

(1) $$g_{j}\left(x_{i}\right)=w_{q}\left(x_{i}\right)$$

where the decision tree’s leaf weights are represented by $w_{q}$. The total of the predictions from each decision tree yields the final forecast for XGBoost:

(2) $${\hat{y}}_{i}=\sum_{j=1}^{M}g_{j}\left(x_{i}\right)$$

where *M* is how many decision trees there are. The objective function in XGBoost is made up of a loss function *l* and a regularization term Ω, which work together to combat the overfitting that decision trees introduce:

(3) $$obj\left(\theta \right)=\sum_{i=1}^{N}l\left(y_{i},{\hat{y}}_{i}\right)+\sum_{j=1}^{M}\Omega \left(f_{i}\right)$$

where *T* is the number of leaves and γ and λ are regularization parameters, and Ω(f) = $\gamma T+\frac{\lambda }{2}$$\sum_{l=1}^{T}w_{l}^{2}$. XGBoost iteratively incorporates new decision trees while training. The *t*th iteration’s prediction is given as

(4) $${\hat{y}}_{i}^{\left(t\right)}={\hat{y}}_{i}^{\left(t-1\right)}+g_{t}\left(x_{i}\right)$$

In accordance with this, the *t*th iteration’s objective function is

(5) $$obj^{\left(t\right)}=\sum_{i=1}^{N}l\left(y_{i},{\hat{y}}_{i}^{\left(t-1\right)}+g_{t}\left(x_{i}\right)\right)+\Omega \left(f_{i}\right)$$

XGBoost presents the loss function’s first and second derivatives. The objective function of the *t*th iteration can be stated as follows by using Taylor expansion on the objective function of the second order:

(6) $$obj^{\left(t\right)}≃\sum_{i=1}^{N}[l(y_{i},{\hat{y}}_{i}^{\left(t-1\right)}+\partial_{{\hat{y}}_{i}\left(t-1\right)}l\left(y_{i},{\hat{y}}_{i}^{\left(t-1\right)}\right)f_{t}\left(x_{i}\right)+\frac{1}{2}\partial_{{\hat{y}}_{i}\left(t-1\right)}^{2}l\left(y_{i},{\hat{y}}_{i}^{\left(t-1\right)}\right)f_{t}^{2}\left(x_{i}\right)+\Omega \left(f_{i}\right)$$

XGBoost can predict the labels of sample data with proportional probabilities. The likelihood that an anonymous patient has breast cancer or not is output by XGBoost in our study. If the estimated chance is greater than 50%, this classification is marked as positive (breast cancer), and if not, it is marked as non-breast cancer.

### 3.4. Selection Model

This study aims to explain how an algorithm discriminates between patients diagnosed with breast cancer and healthy ones. We used a dataset of reproductive-related breast cancer risk factors, a high-fat diet, and body mass index (Nindrea et al. [84]). Through benchmarking, we evaluate different classification algorithms and select the one with the best performance to interpret its results. The performance of the algorithms is measured in terms of accuracy, precision, and recall.

(7) $$accuracy=\frac{TP+TN}{TP+TN+FP+FN}$$

(8) $$precision=\frac{TP}{TP+FP}$$

(9) $$recall=\frac{TP}{TP+FN}$$

where *TP*, *FP*, *FN*, and *TN* correspond to true positives, false positives, false negatives, and true negatives, respectively. True positives (*TP*) are the number of cancer patients correctly identified by the algorithm; false positives (*FP*) are the number of healthy patients that the algorithm incorrectly classifies as having cancer; false negatives (*FN*) are the number of cancer patients who are incorrectly classified as healthy; and true negatives (*TN*) are the number of healthy patients correctly classified. Accuracy in Equation (7) is the ratio that represents the total number of patients correctly classified over the total number of patients analyzed. For breast cancer patients, in this study, the precision in Equation (8) is the ratio that represents the patients with breast cancer correctly identified over the total that the algorithm indicates have cancer. Recall that Equation (9) corresponds to the ratio of correctly identified breast cancer patients out of all cancer patients.

The algorithms selected for benchmarking are logistic regression, random forest, XGBoost, and support vector machine. We chose these algorithms because they are commonly used in classification problems and have been used in previous breast cancer screening studies (see, e.g., Liu [90], Khandezamin et al. [91], Sultana and Jilani [92], Nguyen et al. [93], Begum et al. [94], Kabiraj et al. [95], Mahesh et al. [96], Liew et al. [97], Kim et al. [98], Wang et al. [99], Chiu et al. [100], Alshutbi et al. [101]).

The dataset is divided into 75% training and 25% testing. On the training set, the values of the hyperparameters that maximize accuracy are determined, and these parameters are obtained through a random search, this strategy has proven more efficient for hyperparameter optimization, obtaining better models in less time than a grid search (Shekhar et al. [102]). Appendix A shows the hyperparameter search space for each algorithm. This study used the classification algorithms’ accuracy as a performance measure because the dataset is balanced. That is, the number of cancer patients and healthy patients is similar.

### 3.5. SHAP Mathematical Method: Strategy to Interpret the XGBoost Model of Breast Cancer

The algorithm that obtains the best ranking is selected to interpret its results. We use Shapley Additive Explanations because they are widely used for interpreting machine learning models (Keren Evangeline et al. [103], Zhang et al. [104], Meshoul et al. [105], Larasati [106], Kim et al. [107]). SHAP is derived from game theory and is useful for explaining any ML algorithm. To interpret the model, a reference value is used, and the marginal contribution of each variable to the final result is calculated.

For our model, and also proposed by Lundberg and Lee [108], the prediction function is f(x), and *F* is the set of all input parameters; the SHAP values are obtained as follows:

(10) $$\Phi_{i}=\sum_{S\subseteq F\backslash \{i\}}\frac{|S|!(|F|-|S|-1)!}{|F|!}\left[f_{S\cup \{i\}}\left(x_{S\cup \{i\}}\right)-f_{S}\left(x_{S}\right)\right];$$

where |F| is the number of input parameters of the model, *S* is a subset of features that does not include the ith feature, |S| is the cardinality of this subset, y $f_{s}\left(\right)$ represents the prediction function of the model.

The experiments were carried out in Python to implement the algorithms and search for hyperparameters, and the sklearn and XGBoost libraries were obtained. For the interpretation of the results, the SHAP library was obtained.

## 4. Results

The main results of the methodology used in this research are based on the selection of algorithms, the explainable model, and the interpretation of the prediction at the patient level as a clinical decision support model and actions preventing breast cancer from helping patients.

### 4.1. Algorithm Selection

We have selected the XGBoost algorithm (see Table 1) since it is the model that represents the best performance in terms of precision when compared to the other three ML models. A random search determined the hyperparameters. Table 1 shows the mean values of accuracy, precision, and recall using cross-validation, with k = 10.

Table 1 shows that XGboost presents the best performance in terms of precision in the test dataset and does not lose predictive capacity compared to the result obtained in the train dataset, with the expected result confirming the absence of overfitting. For this reason, we used XGBoost as the classification algorithm to interpret. Table 2 presents the accuracy, precision, and recall over the entire set of tests.

The calculated hyperparameters for XGBoost are as follows: (1) reg lambda = 0.1; (2) reg alpha = 1; (3) n estimators = 400; (4) min child weight = 1; (5) max depth = 3; (6) learning rate = 0.1; (7) gamma = 0.6; (8) colsample bytree = 0.7.

### 4.2. Model Explainability

After selecting the XGBoost algorithm, we have considered providing additional information to physicians and management teams through an interpretability algorithm, which helps explain how the model classified patients.

**Figure 2:**
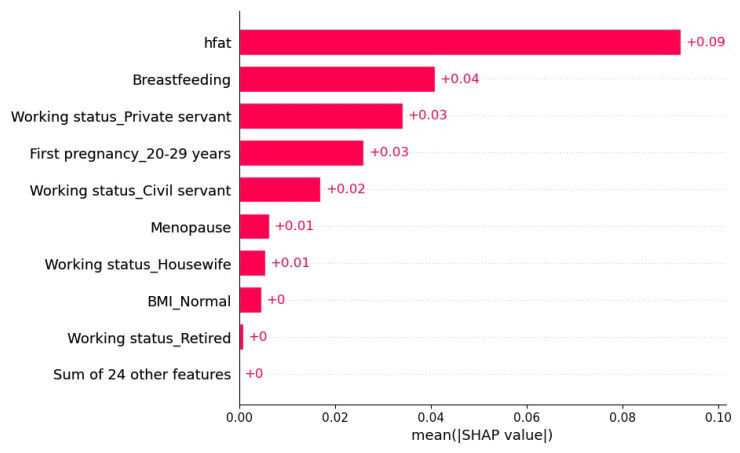
Visualization of explainability variables provided by the SHAP algorithm.

**Figure 3:**
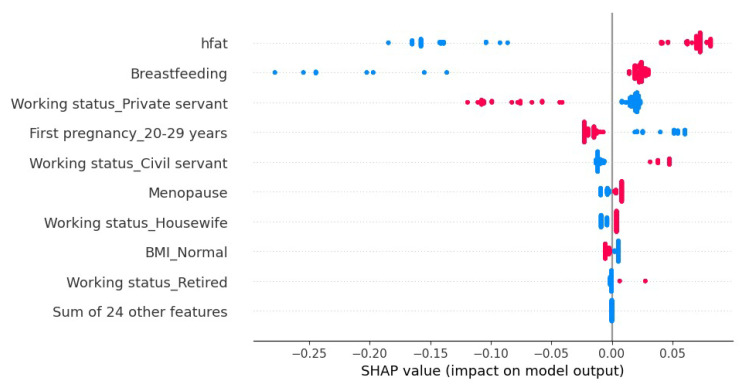
Contribution of each variable for the entire dataset.

Figure 2 represents the extraction of the most significant variables from the XGBoost model for the available patient data. It is observed that the variables high-fat diet and breastfeeding described as hfat y Breastfeeding are the most important variables that allow the algorithm to discriminate between healthy patients and those with breast cancer in Indonesian women. In Figure 3, the blue color represents the absence of the characteristic and red its presence; in this, we can observe that the presence of a high-fat diet has a positive contribution to the prediction of cancer patients, while the absence of a high-fat diet contributes negatively to the prediction. In this way, the XGBoost model mixed with the SHAP interpretability algorithm offers more information for the decision making of health teams.

### 4.3. Interpretation of the Prediction at the Patient Level

It is crucial to comprehend how a model predicts an outcome. In this case, if the XGBoost output is greater than or equal to 0.5, the patient will be classified as having breast cancer, and if the output is less than 0.5, the patient will be classified as having no breast cancer. To interpret the XGBoost prediction, we obtained the SHAP values from the training data. For example, we randomly selected two patients the algorithm correctly classified as not having breast cancer and two correctly classified as having breast cancer.

**Figure 4:**
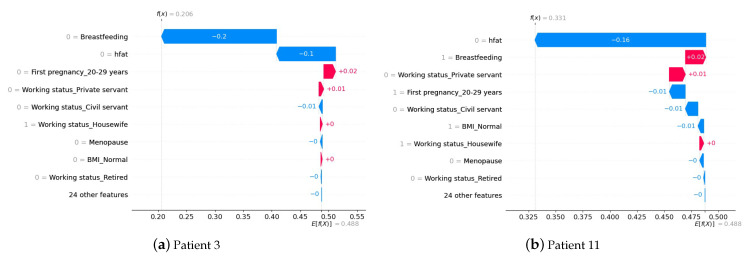
Patients without breast cancer and correctly classified by XGBoost.

Figure 4 shows the patients without breast cancer. The blue color decreases the value of the algorithm’s output, while the red color contributes to increasing the output value. Figure 4a corresponds to patient 3, who had her first pregnancy after 29, i.e., a SHAP value of +0.02, corresponding to the marginal contribution over the reference value (0.488). The positive sign implies that this condition does not satisfy the output value of the algorithm. However, she does not have a high-fat diet and has breastfed for less than one year; together, they have a SHAP value of −0.3 (−0.2 and −0.1, respectively), leaving the algorithm output below the threshold, finally, implying a classification without cancer. Similarly, Figure 4b shows patient 11, who, having breastfed for over a year, will increase the algorithm’s output with a SHAP value of +0.02. However, not having a high-fat diet has a greater impact, and a SHAP value of −0.16, leaving the algorithm output below the threshold, ultimately implying a cancer-free classification.

**Figure 5:**
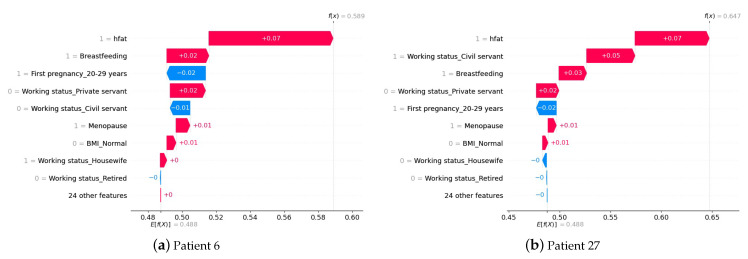
Breast cancer patients correctly classified by XGBoost.

Figure 5 shows the patients with breast cancer. Figure 5a corresponds to patient 6, who, despite having her first pregnancy between the ages of 20 and 29, with a SHAP value of −0.02, the fact of maintaining a high-fat diet and breastfeeding for longer periods at one year has SHAP values of +0.07 and +0.02, respectively, implying that the output of the algorithm is higher than the threshold, classifying the patient with cancer. Similarly, Figure 5b shows patient 27, who, despite having a pregnancy between 20 and 29 years of age, has a SHAP value of −0.02, having a high-fat diet, working as a servant civil, and breastfeeding for periods longer than one year (i.e., SHAP value of +0.15 = +0.07, +0.05 and +0.03, respectively), implying that the output of the algorithm was higher than the threshold, classifying the patient with breast cancer.

## 5. Discussion

The main advantage of the strategy proposed in this research is the chance to interpret the results offered by the combination of ML and XAI algorithms and the knowledge available to health teams when making decisions on breast cancer prevention. Along the same line, the proposed strategy helps patients, in a personalized way, to know the relevant variables and how these variables could increase the risk of suffering from the disease.

The results show a simple method to support the clinical decisions, which allows each case to know precisely the relevant variables of breast cancer prevention. First, we compared different classification algorithms with patients with and without breast cancer. We chose the XGBoost algorithm from this process since it represents better mean accuracy, using cross-validation with k = 10. We subsequently optimized the parameters of the XGBoost algorithm and linked it with the SHAP algorithm. The mix of ML + XAI provided a simple and interpretable method. This method makes it possible to classify new patients at risk of suffering from breast cancer based on a list of variables. The health team and decision makers can analyze the category assigned to a patient with or without breast cancer and understand which rules and the degree of importance determine the result provided by the prediction model.

The structure of the methodology and other relevant elements could evolve, such as technology, environmental conditions, and population size. Therefore, it is necessary to update the methodology since the elements and components of decision making and management that may be affecting the diagnostic opportunity for the care and treatment of patients at risk of developing breast cancer may not be so tomorrow.

According to the WHO, 1 in 12 patients develop breast cancer in their lifetime, 8.3%. This reality is representative of the universe of patients with this health condition. Along the same lines, the raw data available from Indonesian patients to carry out this study were 400 cases, of which 50% were classified as having breast cancer. The balance of cases with a confirmed diagnosis of breast cancer (200 cases) undoubtedly affected the performance of the XGBoost classification model (average accuracy of 0.81 for test data), and we believe that in distributions of cases similar to the registries of WHO, the model could offer better results. The implications of the 15% error in predicting healthy patients should be observed since, in practice, this means leaving patients who require it without treatment.

As a matter of fact, and if this methodology is reproduced and scaled in other health services around the world, it is necessary to consider greater availability of anonymous data from participating patients (i.e., clinical and non-clinical information) for the entire experimentation process (development of strategies of ML and XAI), the participation of health teams, and the resources necessary for development.

Some works in the literature show the impact of COVID-19 in patients with breast cancer (Osareh and Shadgar [21], Ahmad et al. [22], Yue et al. [23]). Unlike these jobs, our hybrid ML + XAI strategy allows health teams to work in a coordinated and collaborative manner, favoring decision making and personalized care.

Considering a breast cancer control mechanism and prevention in women is essential. Although this strategy makes it possible to classify patients, we also suggest an order or ranking of patients with major risks to be cared for earlier by the clinical team (see, e.g., Silva-Aravena et al. [109]). For this reason, we suggest that hospitals in Indonesia do a computerized medical protocol with expert supervision, adding this methodology as support.

When starting the implementation of this methodology, we recommend that health services include additional management components to ensure proper classification and treatment of patients with breast cancer. Despite the results obtained, we suggest that the method can be analyzed and adapted to the particular requirements and needs of hospitals where it is implemented.

## 6. Conclusions

In this research, we follow the standard data management structure in the healthcare field, CRISP-ML(Q), that combines with the SHAP explainability algorithm to develop a hybrid ML strategy to classify anonymous breast cancer patients in Indonesia. The method proposes a novel algorithm that measures some variables, classifies the status of patients, and decides if patients have breast cancer, helping patients who do not have the disease with prevention strategies suggested by the clinical team. The methodology is easy to apply and can help the Indonesian medical team complement their medical decision.

Our work used a universe of 400 anonymous patient cases, 200 of them with breast cancer in Indonesia. The methodology proposes to use different algorithms for classifying and selecting the best according to performance indicators, such as accuracy, recall, and precision. Additionally, the methodology proposed in this work provides new management elements and an explainable machine learning strategy through the SHAP algorithm that offers better quality information to health specialists to make decisions based on data about patients at risk of developing breast cancer.

The resulting model offered by the approach is the ease of interpreting the classification of patients with and without breast cancer. The interpretability strategy helps patients and the health team with strategies and suggestions for preventing the disease since it allows timely knowledge of which variable, in what way, and with what level of quantitative importance could affect each patient individually. The strategy proposed in this paper identifies two variables, high-fat diet, and breastfeeding, as the most relevant when classifying patients in the clinical evaluation process.

In future work, we suggest analyzing the imbalance of cases observed in healthy patients and those with breast cancer in the real world.

To implement the methodology in this research in hospitals worldwide, we suggest that health centers have all the relevant information in the decision-making process about patients with and without breast cancer (i.e., data, relevant variables, and clinical aspects and administrative management), which allow, on the one hand, classifying with greater precision and, on the other hand, to validating the management strategy with a higher level of support and participation of the health teams.

## Figures and Tables

**Table 1:** Accuracy, precision, and recall when performing cross-validation, k = 10, in training and test set.

Algorithm	Phase	Label	Precision	Recall	Accuracy
XGBoots	Train	1	91.7%	75.0%	81.33%
		0	71.8%	90.3%	
	Test	1	85.7%	81.4%	81.00%
		0	75.0%	80.5%	
Logistic Regression	Train	1	88.2%	76.5%	81.33%
		0	75.0%	87.3%	
	Test	1	82.1%	78.0%	77.00%
		0	70.5%	75.6%	
Random Forest	Train	1	87.5%	75.9%	80.67%
		0	74.4%	86.6%	
	Test	1	83.9%	79.7%	79.00%
		0	72.7%	78.0%	
SVM	Train	1	89.6%	76.8%	82.00%
		0	75.0%	88.6%	
	Test	1	83.9%	77.0%	77.00%
		0	68.2%	76.9%	

**Table 2:** Accuracy, precision, and recall obtained from XGBoost with the total test data.

Algorithm	Label	Precision	Recall	Accuracy
XGBoots	1	85.4%	79.5%	85.0%
	0	84.7%	89.3%	
Logistic Regression	1	75.0%	81.8%	80.0%
	0	84.6%	78.6%	
Random Forest	1	75.5%	84.1%	81.0%
	0	86.3%	78.6%	
SVM	1	81.0%	77.3%	82.0%
	0	82.8%	85.7%	

## Data Availability

Public records are available in Mendeley Data https://data.mendeley.com/datasets/xfcyrffhy7/2 (accessed on 1 February 2023), and their description in Nindrea et al. [84].

## Appendix A. Hyperparameter Search Space for Each Algorithm

**Algorithm**	**Hyperparameter**	**Search Space**
XGBoots	n_estimators	[100, 200, 300, 400, 500, 600, 700, 800, 900, 1000]
	learning_rate	[0.0001, 0.001, 0.01, 0.1, 1]
	max_depth	range(3, 21, 3)
	min_child_weight	range(1, 21, 3)
	gamma	[i/10.0 for i in range(0, 7)]
	colsample_bytree	[i/10.0 for i in range(3, 10)]
	reg_alpha	[0.00001, 0.01, 0.1, 1, 10, 40, 80, 100]
	reg_lambda	[0.00001, 0.01, 0.1, 1, 10, 40, 80, 100]
Logistic Regression	penalty	[‘l1’, ‘l2’, ‘elasticnet’]
	dual	[True, False]
	tol	[0.0001, 0.001, 0.01, 0.1, 1, 10, 100, 1000]
	C	[0.0001, 0.001, 0.01, 0.1, 1, 10, 100, 1000]
	intercept_scaling	[1, 2, 3, 4, 5]
	solver	[‘newton-cg’, ‘lbfgs’, ‘sag’, ‘saga’]
Random Forest	n_estimators	[5, 20, 50, 100]
	max_features	[‘auto’, ‘sqrt’]
	max_depth	[int(x) for x in np.linspace(10, 120, num = 12)]
	min_samples_split	[2, 6, 10]
	min_samples_leaf	[1, 3, 4]
	bootstrap	[True, False]
SVM	C	[0.1, 1, 10, 100, 1000]
	gamma	[“scale”, “auto”]
	kernel	[‘rbf’, ‘poly’, ‘sigmoid’]
	degree	[1, 2, 3, 4, 5, 6, 7, 8, 9, 10]
	coef0	[0.1, 0.5, 1, 2, 5, 10]
	shrinking	[True, False]
	probability	[True, False]
	tol	[0.0001, 0.001, 0.01, 0.1, 1, 10, 100, 1000]
	cache_size	[200, 500, 1000]
	class_weight	[None, “balanced”]
	decision_function_shape	[‘ovo’, ‘ovr’]
	break_ties	[True, False]
	decision_function_shape	[‘ovo’, ‘ovr’]
	break_ties	[True, False]
